# A nationwide cross-sectional analysis of biopsy-proven Fabry nephropathy: the Japan Renal Biopsy Registry

**DOI:** 10.1007/s10157-022-02287-w

**Published:** 2022-11-03

**Authors:** Makoto Nasu, Naoki Nakagawa, Shigeo Hara, Junko Yano, Yuka Kurokawa, Nao Nakamura, Hitoshi Yokoyama, Akira Shimizu, Hitoshi Sugiyama, Hiroshi Sato, Kei Fukami

**Affiliations:** 1 Division of Nephrology, Department of Medicine, Kurume University School of Medicine, 67 Asahi-machi, Kurume, Fukuoka Japan; 2 Division of Cardiology, Nephrology, Respiratory and Neurology, Department of Internal Medicine, Asahikawa Medical University, Asahikawa, Japan; 3 Department of Diagnostic Pathology, Kobe City Medical Center General Hospital, Kobe, Japan; 4 Department of Nephrology, Kanazawa Medical University School of Medicine, Ishikawa, Japan; 5 Department of Analytic Human Pathology, Nippon Medical School, Tokyo, Japan; 6 Department of Medicine, Kawasaki Medical School General Medical Center and Department of Medical Care Work, Kawasaki College of Allied Health Professions, Okayama, Japan; 7 Department of Internal Medicine, Sendai Hospital of East Japan Railway Company, Sendai, Japan

**Keywords:** Blood pressure, Fabry disease, Fabry nephropathy, Renal biopsy, Screening

## Abstract

**Background:**

Fabry disease (FD) is an X-linked inherited disease where renal complications are associated with a poor prognosis. However, little is known about the prevalence of Fabry nephropathy (FN) in patients with chronic kidney disease (CKD). We extracted FN data from the Japan Renal Biopsy Registry, analyzed the prevalence of FN, and examined the correlation between clinical characteristics and renal involvement according to sex differences and hemi- and heterozygosity in patients with FD.

**Methods:**

A total of 38,351 participants who underwent renal biopsy were retrospectively enrolled, and FN was determined. The clinical characteristics of FD patients were examined based on sex differences.

**Results:**

Twenty-nine patients (0.076%) (19 males and 10 females, mean age: 43.7 ± 15.5 years old) were diagnosed with FN. Median estimated urinary protein (UP) and mean eGFR levels were 0.9 [interquartile range (IQR) [0.7–1.6] g/gCr and 67.1 ± 36.8 mL/min/1.73 m^2^, respectively. Mean systolic blood pressure (SBP) was 126.4 ± 17.1 mmHg and diastolic blood pressure was 76.1 ± 12.6 mmHg. An inverse correlation between eGFR and logarithm UP levels was observed (*r*^2^ = 0.23, *p* = 0.02), SBP was positively associated with logarithm UP (*r*^2^ = 0.34, *p* = 0.004) overall and inversely associated with eGFR (*r*^2^ = 0.25, *p* = 0.007) regardless of sex, and SBP was an independent determinant of proteinuria (*p* = 0.004) and eGFR (*p* = 0.007).

**Conclusions:**

The prevalence of biopsy-proven FN was 0.076%. Since SBP is associated with eGFR regardless of zygosity, strict SBP control might be necessary to prevent progression to end-stage kidney disease in both male and female patients with FN.

**Supplementary Information:**

The online version contains supplementary material available at 10.1007/s10157-022-02287-w.

## Introduction

Fabry disease (FD) is an inborn metabolic error caused by a deficiency of alpha-galactosidase A (GLA), a lysosomal hydrolytic enzyme that is inherited in an X-linked manner [1, 2]. The accumulation of globotriaosylceramide (Gb3), which cannot be degraded in various cells throughout the body, causes tissue and organ damage in the kidney, heart, and cerebrovascular tissues, thereby reducing the life expectancy of patients with FD [3]. 

The prevalence of FD in Europe and the United States was initially reported to be approximately 1/40,000, but a screening test of newborn infants showed that the number of boys with low GLA activity was approximately 1/3100 in Italy [4] and 1/1368 in Taiwan [5]. In Japan, a pilot study of a newborn mass screening conducted by Kumamoto University and Fukuoka University reported that three out of 21,170 newborns were diagnosed with FD, a higher prevalence than previously reported (1/7057 in all babies; 1/3609 in boys only) [6]. In adults, the prevalence of male FD patients with end-stage kidney disease (ESKD) undergoing hemodialysis, left ventricular hypertrophy or hypertrophic cardiomyopathy, and acute subclinical stroke was 0.25–1%, 1.0%, and 5%, respectively, as examined by plasma GLA activity [7–10]. Furthermore, we recently reported that the prevalence of FD in male patients with chronic kidney disease (CKD) with and without dialysis is 0.06% (1/1703)–0.48% (2/419), respectively [11].

To date, FD screening for evaluating the prevalence of Fabry nephropathy (FN) in male patients with CKD is measured by GLA activity; however, due to the lack of a precise diagnosis of FN, other kidney diseases might coexist in FD patients with renal involvement. Furthermore, female patients are not suitable for screening due to residual GLA activity. Therefore, renal biopsy is necessary to examine the precise prevalence of FN in male and female patients with CKD. In this study, we investigated the prevalence of renal biopsy-proven FN in patients with CKD registered in the Japan Renal Biopsy Registry (J-RBR), organized by the Japanese Society of Nephrology. Further, we evaluated the clinical characteristics of FN according to sex differences (hemizygous and heterozygous).

## Materials and methods

### Enrollment of FN patients

**Fig. 1:**
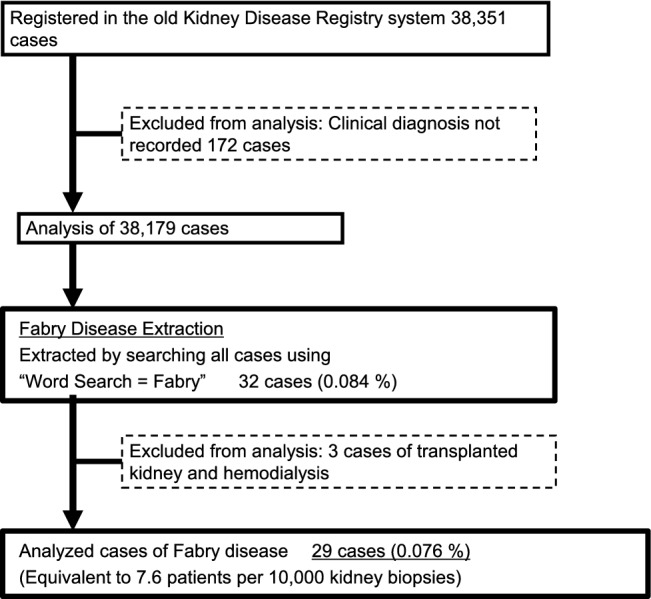
Flow chart of the extraction of Fabry nephropathy from J-RBR

Of a total of 38,351 registered patients who underwent renal biopsy between July 2007 and January 2018 in the J-RBR, 172 patients with undescribed clinical diagnoses were excluded, and the remaining 38,179 patients were included in this study (Fig. 1). The clinical characteristics of the patients, including age, sex, laboratory data, and physiological and pathological parameters, including proteinuria (g/gCr), hematuria, estimated glomerular filtration rate (eGFR), systolic blood pressure (SBP), diastolic blood pressure (DBP), and antihypertensive drug use, were recorded on the J-RBR website using the Internet Data and Information Center for Medical Research system of the University Hospital Medical Information Network (UMIN) [12]. The J-RBR has been registered under the Clinical Trial Registry of the UMIN (registration number: UMIN000000618). We collected data of FN by "Word search = Fabry,” and examined the correlation among parameters according to zygosity difference.

eGFR was calculated using the following formula:

- For adults:
  $${\text{eGFR }}\left( {{\text{mL}}/\min /1.73{\text{m}}^{{2}} } \right) = 194 \times {\text{sCr}}^{ - 1.094} \times {\text{age}}^{ - 0.287} \times [0.739 + 0.261 \times ({\text{sex}} - 1)].$$

Sex male = 2, female = 1 [13, 14].

- For pediatrics (under 18 years of age):
  $${\text{Female child ref}}:{\text{ Cr}} = - 4.536{\text{Ht}}^{{5}} + 27.16{\text{Ht}}^{{4}} - 63.47{\text{Ht}}^{{3}} + 72.43{\text{Ht}}^{{2}} - 40.06{\text{Ht}} + 8.778,$$
  $${\text{Male child ref: Cr}} = - 1.259{\text{Ht}}^{{5}} + 7.815{\text{Ht}}^{{4}} - 18.57{\text{Ht}}^{{3}} + 21.39{\text{Ht}}^{{2}} - 11.71{\text{Ht}} + 2.628,$$
  $${\text{eGFR }}\left( {{\text{mL}}/\min /1.73{\text{m}}^{{2}} } \right) = 110.2 \times \left( {\text{ref Cr/sCr}} \right) + 2.93.$$

Ht = height (m), sCr = serum creatinine [15].

### Statistical analysis

Results are presented as the median or mean ± standard deviation. To perform linear regression analysis, UP was log-transformed after adding 0.1. Univariate regression analysis was performed to determine the correlations between eGFR and proteinuria, proteinuria and SBP, and eGFR and SBP. Multiple regression analysis was performed to assess independent determinants of eGFR and proteinuria. Differences between the groups were assessed using the chi-squared test or unpaired *t* test. All statistical analyses were performed using the JMP Pro ver. 15 software (SAS Institute, Inc.). Statistical significance was set at *P* < 0.05.

## Results

### Prevalence of Fabry nephropathy

Of the 38,179 enrolled patients with CKD, 32 (0.084%) were classified as patients with FD who were not treated with enzyme replacement therapy (ERT). Three participants with FD were excluded because they had undergone renal transplantation or hemodialysis. Finally, 29 cases were analyzed, and the prevalence of FN was 0.076%, equivalent to 7.6 patients per 10,000 kidney biopsies (Fig. 1).

### Clinical characteristics in patients with Fabry nephropathy

The clinical characteristics of all the patients with FN are shown in Tables 1, 2. Of the 29 patients with FN, 19 (65.5%) were males (hemizygous) and 10 (34.5%) were females (heterozygous). Mean age was 43.7 ± 15.5 years (males: 42.2 ± 17.7 years, females: 46.5 ± 10.4 years); two males were under 18 years of age. Mean SBP and DBP were 126.4 ± 17.1 mmHg and 76.1 ± 12.6 mmHg, respectively. The hematuria positivity rate was 31.0% (*n* = 9) in all patients. Mean eGFR was 67.1 ± 36.8 mL/min/1.73 m^2^, and median estimated proteinuria was 0.9 [IQR 0.7–1.6] g/gCr. A total of 56% of patients (14 out of 25) and 13.6% of patients (three out of 22) were prescribed antihypertensive drugs and had a history of diabetes, respectively (Tables 1, 2).

**Table 1:** Clinical characteristics of the FD patients

No	Sex	Age (years)	BMI	SBP (mmHg)	DBP (mmHg)	TP (g/dL)	sALB (g/dL)	TC (mg/dL)	sCr (mg/dL)	eGFR(mL/min/1.73m^2^)	UP/UCrea (g/gCr)	HU	Antihypertensive drug	History of DM
1	F	37	23.0	114	67	6.2	3.8	182	0.5	101.9	0.2	( +)	−	−
2	F	41	22.5	110	50	7.6	4.3	170	0.9	54.1	N/A	( +)	+	−
3	M	17	29.9	145	85	7.5	4.6	189	0.6	155.4	N/A	( − )	N/A	N/A
4	M	49	18.0	143	91	7.1	4.1	193	1.9	31.5	0.9	( +)	+	−
5	F	67	15.0	140	80	7.5	4.3	178	1.9	20.9	3.0	( − )	+	+
6	M	36	19.8	110	68	7.3	4.5	171	0.8	92.3	0.0	( − )	−	−
7	M	38	24.7	116	71	6.3	3.9	203	1.1	63.4	0.9	( − )	+	−
8	M	55	23.5	124	70	5.9	3.4	220	0.9	68.9	N/A	( +)	+	−
9	M	33	26.1	123	88	7.0	4.3	312	0.8	90.8	1.9	( − )	−	−
10	M	68	27.2	146	83	7.3	4.5	215	1.6	35.8	1.1	( − )	+	+
11	F	41	20.3	110	58	6.8	3.8	246	0.7	71.8	0.9	( − )	+	−
12	M	56	28.7	117	81	6.5	3.7	192	1.1	56.7	0.3	( − )	+	+
13	F	39	21.0	N/A	N/A	6.7	2.8	258	0.7	74.0	4.7	( +)	N/A	N/A
14	M	13	14.5	99	56	6.9	4.2	139	0.4	170.8	N/A	( +)	−	N/A
15	M	60	21.1	108	86	6.9	4.0	238	0.9	66.4	0.8	( − )	−	−
16	M	56	18.1	131	65	6.9	4.0	163	2.8	19.9	1.3	( − )	+	−
17	F	34	22.1	115	75	7.0	4.0	203	0.7	82.1	0.5	(−)	−	−
18	F	47	23.9	122	72	6.6	4.0	210	0.7	70.1	0.5	( − )	−	−
19	M	27	23.2	129	83	7.4	4.2	180	0.8	98.9	0.3	( +)	−	−
20	M	25	25.8	182	110	6.3	3.8	221	6.6	9.8	2.0	( − )	N/A	N/A
21	M	34	20.2	125	78	6.8	4.1	153	1.6	43.0	N/A	( − )	+	−
22	M	35	21.1	142	93	6.5	3.8	167	1.1	65.6	2.3	( − )	N/A	N/A
23	F	59	21.6	123	71	6.9	4.5	205	0.7	62.8	0.2	( +)	−	−
24	M	66	22.6	131	73	6.4	3.9	182	1.6	34.9	0.3	( − )	+	−
25	M	72	18.9	154	90	6.5	3.4	288	2.0	26.3	3.9	( +)	+	N/A
26	M	26	22.2	117	70	6.9	4.0	N/A	0.8	102.8	N/A	( − )	−	−
27	F	49	23.9	120	64	7.2	4.3	220	0.7	72.7	0.1	( − )	−	−
28	F	51	20.5	121	76	7.7	4.2	N/A	0.7	65.4	0.1	( − )	+	−
29	M	35	30.5	123	77	6.0	3.2	155	1.8	37.4	1.2	( − )	+	N/A

*BMI* body mass index, *DBP* diastolic blood pressure, *DM* diabetes mellitus, *eGFR* estimated glomerular filtration rate, *FD* Fabry disease, *HU* hematuria, *sALB* serum albumin, *SBP* systolic blood pressure, *sCr* serum creatinine, *TC* total cholesterol, *TP* total protein, *UP/UCrea* urinary protein/urinary creatinine ratio

**Table 2:** Difference of clinical characteristics with sex in FD patients

	All	Number	Males	Number	Females	Number	*P* value
Age	43.7 ± 15.5	*n* = 29	42.2 ± 17.7	*n* = 19	46.5 ± 10.4	*n* = 10	0.41
BMI	22.4 ± 3.9	*n* = 29	23.0 ± 4.3	*n* = 19	21.4 ± 2.6	*n* = 10	0.23
SBP (mmHg)	126.4 ± 17.1	*n *= 28	129.7 ± 19.2	*n* = 19	119.4 ± 9.2	*n* = 9	0.07
DBP (mmHg)	76.1 ± 12.6	*n* = 28	79.9 ± 12.3	*n* = 19	68.1 ± 9.5	*n* = 9	0.01
TP (g/dL)	6.8 ± 0.5	*n* = 29	6.8 ± 0.5	*n* = 19	7.0 ± 0.5	*n* = 10	0.18
sALB (g/dL)	4.0 ± 0.4	*n* = 29	4.0 ± 0.4	*n* = 19	4.0 ± 0.5	*n* = 10	0.90
TC (mg/dL)	202.0 ± 40.5	*n* = 27	198.9 ± 45.4	*n* = 18	208.0 ± 29.9	*n* = 9	0.54
sCr (mg/dL)	0.9 [IQR 0.7–1.6]	*n* = 29	1.1 [IQR 0.8–1.8]	*n* = 19	0.7 [IQR 0.7–0.8]	*n* = 10	0.05
eGFR (mL/min/1.73m^2^)	67.1 ± 36.8	*n* = 29	66.9 ± 43.5	*n* = 19	67.6 ± 20.7	*n* = 10	0.95
UP/UCrea (g/gCr)	0.9 [IQR 0.3–1.9]	*n* = 23	1.0 [IQR 0.3–1.9]	*n* = 14	0.5 [IQR 0.2–1.9]	*n* = 9	0.89
HU	Positive *n* = 9 (31.0%) Negative *n* = 20 (69.0%)	*n* = 29	Positive n = 5 (26.3%) Negative *n* = 14 (73.7%)	*n* = 19	Positive *n* = 4 (40%) Negative *n* = 6 (60%)	*n* = 10	0.67
Antihypertensive drugs	( +) *n* = 14 (56%) ( − ) *n* = 11 (44%)	*n* = 25	( +) *n* = 10 (63%) ( − ) *n* = 6 (37%)	*n* = 16	( +) *n* = 4 (44%) ( − ) *n* = 5 (56%)	*n* = 9	0.43
History of DM	( +) *n* = 3 (13.6%) ( − ) *n* = 19 (86.4%)	*n* = 22	( +) *n* = 2 (15.4%) ( − ) *n* = 11 (84.6%)	*n* = 13	( +) *n* = 1 (11.1%) ( − ) *n* = 8 (88.9%)	*n* = 9	1

Underline indicates *P*<0.05

*BMI* body mass index, *DBP* diastolic blood pressure, *DM* diabetes mellitus, *eGFR* estimated glomerular filtration rate, *FD* Fabry disease,* HU* hematuria, *sALB* serum albumin, *SBP* systolic blood pressure, *sCr* serum creatinine, *TC* total cholesterol, *TP* total protein*, UP/UCrea* urinary protein/urinary creatinine ratio. Numbers are mean ± standard deviation or median [interquartile range (IQR)]

### Number and rate of patients according to the CKD classification by gender difference

**Fig. 2:**
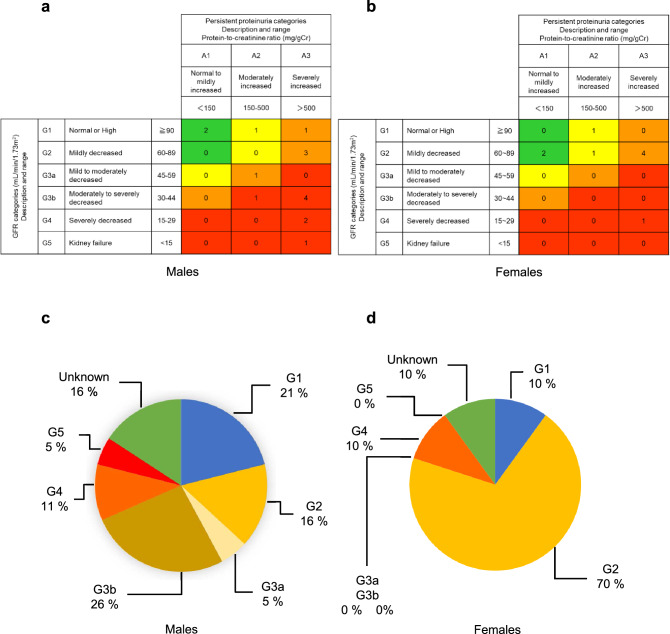
Number and rate of patients according to the CKD classification by gender difference. Number of male (**a**) and female (**b**) patients according to classification. Rate of patients according to the classification of males (**c**) and females (**d**) CKD, chronic kidney disease; GFR, glomerular filtration rate

The classification of CKD stage based on eGFR and estimated UP levels at kidney biopsy showed that G1: *n* = 4 (21%), G2: *n* = 3 (16%), G3a: *n* = 1 (5%), G3b:* n* = 5 (26%), G4: *n* = 2 (11%), G5: *n* = 1 (5%), and unclassifiable: *n* = 3 (16%) in men and G1: *n* = 1 (10%), G2: *n* = 7 (70%), G3a: *n* = 0 (0%), G3b: *n* = 0 (0%), G4: *n* = 1 (10%), and unclassifiable: *n* = 1 (10%) in women (Figs. 2a–d). No difference in mean eGFR between men and women was observed (Table 2); however, some male FN patients showed more severe CKD classification than female FN patients (Figs. 2a–d).

### Difference in proteinuria, eGFR, hematuria, and blood pressure according to gender differences in patients with Fabry nephropathy

**Fig. 3:**
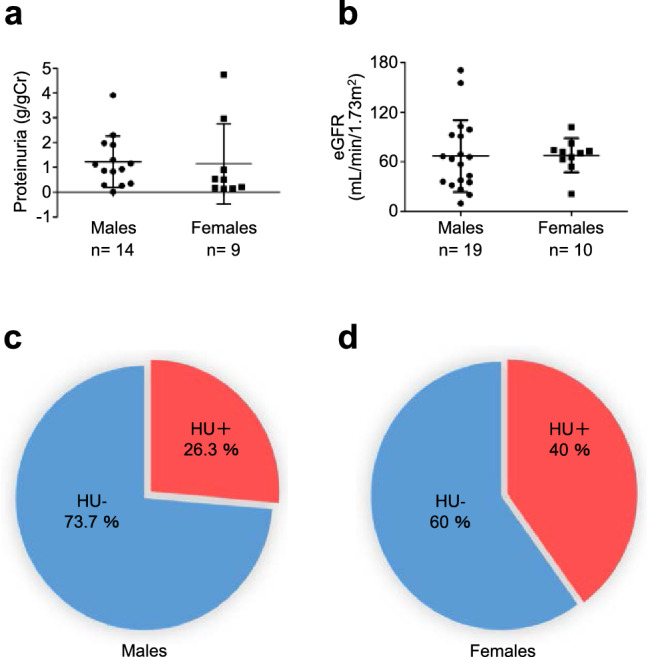
Difference of the prevalence of proteinuria, eGFR levels, and hematuria according to sex difference. Differences in proteinuria (**a**), eGFR (**b**), and hematuria (**c**) according to sex eGFR, estimated glomerular filtration rate; HU, hematuria

Median proteinuria level was 1.1 [IQR 0.8–1.8] g/gCr for males (*n* = 14) and 0.7 [IQR 0.7–0.8] g/gCr for females (*n* = 9), with no significant difference (*p* = 0.89) (Table 2 and Fig. 3a). Mean eGFR was 66.9 ± 43.5 mL/min/1.73 m^2^ for males (*n* = 19) and 67.6 ± 20.7 mL/min/1.73 m^2^ for females (*n* = 10), with no significant difference (*p* = 0.95) (Table 2 and Fig. 3b). The prevalence of hematuria was 26.3% and 40% in men and women, respectively, with no significant difference (*p* = 0.67) (Table 2, Fig. 3c, d). Mean SBP tended to be higher in males compared with that in females (males: 129.7 ± 19.2 mmHg, *n* = 19, females: 119.4 ± 9.2 mmHg, *n* = 9, *p* = 0.07), and mean DBP was significantly higher in males compared with that in females (males: 79.9 ± 12.3 mmHg, *n* = 19, females: 68.1 ± 9.5 mmHg, *n* = 9, *p* = 0.01) (Table 2).

### Correlation between proteinuria and eGFR, and determinants of eGFR in patients with Fabry nephropathy

**Fig. 4:**
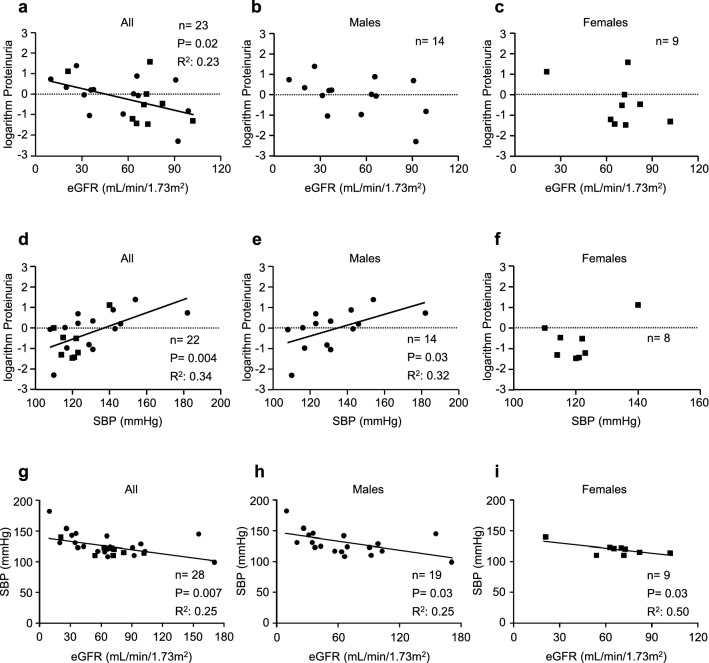
Correlation between eGFR and logarithm proteinuria in all patients (**a**), males (**b**), and females (**c**). Correlation between logarithm proteinuria and SBP in all patients (**d**), males (**e**), and females (**f**). Correlation between eGFR and SBP in all patients (**g**), males (**h**), and females (**i**). eGFR, estimated glomerular filtration rate; SBP, systolic blood pressure

**Fig. 5:**
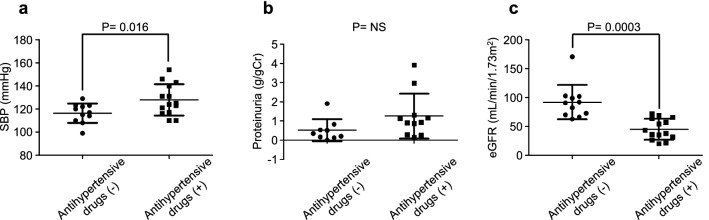
Comparison of SBP, proteinuria, and eGFR between antihypertensive drug users and nonusers SBP (**a**), proteinuria (**b**), and eGFR (**c**) eGFR, estimated glomerular filtration rate, SBP, systolic blood pressure

There was a significant correlation between eGFR and logarithm UP levels in all FN patients (*n* = 23, *r*^2^ = 0.23, *p* = 0.02); however, no correlation by gender (Fig. 4a–c). Overall, an inverse correlation between eGFR and logarithm UP levels was observed (*r*^2^ = 0.23, *p* = 0.02) (Fig. 4a). Systolic BP was positively associated with logarithm UP in all (*r*^2^ = 0.34, *p* = 0.004) and male (*r*^2^ = 0.32, *p* = 0.03) FN patients, however, not in females (Fig. 4d–f). An inverse association between SBP and eGFR in all (*r*^2^ = 0.25, *p* = 0.007), males (*r*^2^ = 0.25, *p* = 0.03), and females (*r*^2^ = 0.50, *p* = 0.03) was observed (Fig. 4g–i). Univariate and multivariate regression analyses were performed to examine independent determinants of proteinuria and eGFR. Serum albumin (inversely), total cholesterol (positively), and SBP (positively) were correlated with UP levels, while SBP was an independent determinant of UP level (*P* = 0.003) (Table 3). Similarly, SBP was the sole independent determinant of eGFR in patients with FN (Table 4). Finally, we compared SBP, proteinuria, and eGFR between antihypertensive drug users and nonusers. SBP was higher (127.9 ± 13.6, 116.4 ± 8.4 mmHg, respectively, *p* = 0.016) and eGFR was lower (45.0 ± 18.1, 92.0 ± 29.9 mL/min/1.73 m^2^, respectively, *p* = 0.0003) in antihypertensive drugs users than those in nonusers (Fig. 5a, c). Urinary protein levels tended to be higher in antihypertensive users than in nonusers (1.3 ± 1.2, 0.5 ± 0.6 g/gCr, respectively, *p* = 0.08) (Fig. 5b).

**Table 3:** Univariate and multivariate regression analyses for the determinants of proteinuria

Variables	Univariate regression			Multivariate regression		
	SE	*β*	*p*	SE	*β*	*p*
Age	0.020	0.099	0.655			
Sex	0.275	0.033	0.882			
BMI	0.073	− 0.268	0.216			
**SBP**	**0.010**	**0.623**	**0.002**	**0.009**	**0.558**	**0.003**
**sALB**	**0.549**	**− 0.576**	**0.004**	0.489	− 0.255	0.138
**TC**	**0.006**	**0.455**	**0.033**	0.004	0.335	0.056
eGFR	0.010	− 0.340	0.112			
Hematuria	0.297	0.247	0.256			
History of DM	0.218	− 0.441	0.067			

Bold indicates *P*<0.05

*BMI* body mass index, *DM* diabetes mellitus, *eGFR* estimated glomerular filtration rate, *sALB* serum albumin, *SBP* systolic blood pressure, *TC* total cholesterol, *UP/UCrea* urinary protein/urinary creatinine ratio

**Table 4:** Univariate and multiple regression analyses for the determinants of eGFR

Variables	Univariate regression			Multiple regression		
	SE	*β*	*p*	SE	*β*	*p*
Sex	7.324	− 0.009	0.962			
BMI	1.837	0.034	0.860			
**SBP**	**0.371**	**− 0.501**	**0.007**	**0.371**	**− 0.501**	**0.007**
sALB	16.832	0.270	0.156			
TC	0.184	0.113	0.575			
UP/UCrea	4.364	− 0.340	0.112			
Hematuria	7.409	− 0.175	0.363			
History of DM	7.263	0.423	0.050			

Bold indicates *P*<0.05

*BMI* body mass index, *DM* diabetes mellitus, *eGFR* estimated glomerular filtration rate, *sALB* serum albumin, *SBP* systolic blood pressure, *sCr* serum creatinine, *TC* total cholesterol, *UP/UCrea* urinary protein/urinary creatinine ratio

## Discussion

Nephropathy is a major complication in patients with FD, which exacerbates the quality of life and life expectancy. Therefore, screening for FN in patients with CKD is valuable not only for evaluating the prevalence of this disease but also for implementing early exposure to ERT or chaperone therapy to inhibit progression to ESKD. In this retrospective study, we report, for the first time, that the prevalence of biopsy-proven FN in patients with CKD is 0.076%. To date, the prevalence of FD in male patients with CKD has been reported to be 0.48% [11], which is much higher than that reported in our study. These discrepancies may be explained by several factors. First, the screening of FD in previously reported patients with CKD was only performed in a specific region, which might have influenced the high prevalence of FD. Second, due to the residual GLA activity in female (heterozygous) FD patients, patients with pre-dialysis CKD that were enrolled in the previous study for FD screenings were all males [16, 17], and compared with heterozygous mutations, hemizygous mutations are more likely to induce renal dysfunction, which could lead to a high incidence of FN. Third, the number of patients with CKD in previous screening studies was small, and the number of patients with CKD in our study was the largest. Finally, the diagnosis of FD in patients with CKD in previous reports was performed by measuring GLA activity, not by renal biopsy, so other kidney diseases might coexist with FD, which could overestimate the prevalence of FN. Indeed, we have recently reported an FD patient with nephrotic syndrome who achieved complete remission with steroid and immunosuppressant therapy, suggesting the coexistence of focal segmental glomerulosclerosis [18]. Therefore, the prevalence of FN in our study might be more accurate than that reported in previous studies.

The sex difference, in other words, hemizygous and heterozygous mutations, might affect the development of FN. However, in this study, there was no sex-related difference in age, proteinuria, or eGFR levels at the time of renal biopsy. Small sample size with a non-normal distribution of these factors might affect the results. Indeed, the median age was 36 years for males and 44 years for females. Further, 58% (11/19) of the males and 30% (3/10) of the females were under 40 years of age. Thus, it is possible that males are younger than females in this study population; two males over 120 mL/min/1.73 m^2^ were outliers with respect to eGFR, which may affect the mean eGFR for males. Furthermore, it is speculated that different mutation types, such as classical and late-onset gene mutations, might affect these results. Classical mutations in FD accelerate the development of multiple organ damage in young patients, whereas FN with late-onset gene mutations becomes prevalent in elderly patients with both hemi-and heterozygous mutations. In our study, four male patients diagnosed with FN were over 60 years of age, suggesting that they might have the late-onset FD gene mutation. Since there was no information regarding the gene mutation in J-RBR, further verification is needed to clarify this issue. In addition, some patients were positive for hematuria, and the rate of hematuria was higher in FN patients with heterozygous gene mutations than in those with hemizygote mutations. Hematuria is not a specific characteristic feature of FN; therefore, some comorbidities, such as urinary tract infection, menstruation, or both, might result in hematuria. Furthermore, three FN patients were negative for proteinuria (UP/UCrea < 0.15 g/gCr) with normal renal function (eGFR > 60 mL/min/1.73 m^2^) at the time of renal biopsy. A family history of FD, existence of other organ damage, or both, such as cardiac myopathy related to FD, may have been the reason for renal biopsy. Since it has been reported that albuminuria is found only in 55% of male and 35% of female FN patients with an eGFR of 60 mL/min/1.73 m^2^ or higher [19], renal biopsy might be valuable when FD is suspected in normoalbuminuric patients with a specific FD history.

Accumulation of Gb3 in enlarged podocytes is a feature of FN, which impairs podocyte function and accelerates proteinuria, followed by detachment from the glomerular basement membrane, leading to ESKD [20]. Ortiz et al. reported an inverse correlation between proteinuria and eGFR in the Fabry Registry [19]. In the present study, there was a significant correlation between logarithm UP and eGFR, logarithm UP and SBP, and SBP and eGFR in all FN patients. However, especially, there was no correlation in females. Small numbers with different baseline eGFR and proteinuria levels in female patients may affect the results. eGFR and logarithm UP levels correlated with SBP in this study. In multivariate regression analysis, SBP was the sole independent determinant of proteinuria and eGFR, suggesting that SBP might be the most influential factor for renal dysfunction in FN patients. A similar positive correlation between SBP and proteinuria was observed in the Fabry Registry study [19]. Furthermore, the Fabry Outcome Survey suggested a high prevalence of uncontrolled hypertension among patients with FD [21]. They found that uncontrolled hypertension was observed in 57% of male and 47% of female FD patients [21]. They reported that blood pressure control with renin-angiotensin system (RAS) inhibitors was underused among patients with FD who had a high prevalence of impaired kidney function [21]. In our study, only half of the FN patients were prescribed antihypertensive drugs, and their SBP was higher and eGFR was lower. RAS inhibitors are the preferred therapy for hypertension in patients with FD. The European expert consensus statement suggested that ERT alone does not significantly reduce proteinuria, and additional treatment with RAS inhibitors is paramount for reducing proteinuria and the consequent risk of CKD progression [22]. Since a 2 year course of ERT significantly decreased both SBP and DBP [21], antihypertensive drugs in addition to ERT therapy, might reduce the progression of organ damage such as nephropathy, cardiomyopathy, and stroke via strict blood pressure control in patients with FN.

The present study had some limitations: First, the small sample size was insufficient for analysis and rendered it difficult to draw definite conclusions from this study. Second, data on genetic information, symptoms, information on urinary mulberry body, and family history are not available in J-RBR. Third, drug-induced lysosomal disorders cannot be ruled out. Therefore, large numbers of FN patients diagnosed with renal biopsy and genetic analysis with detailed information are needed to clarify which clinical and pathological characteristics are correlated with progression of FN in future.

In conclusion, the prevalence of biopsy-proven FN is 0.076% in Japan. Since SBP is associated with eGFR and proteinuria regardless of sex, strict SBP control might be necessary to prevent the progression to ESKD in both male and female patients with FN.

## Supplementary Information

Below is the link to the electronic supplementary material.

Supplementary file1 (DOCX 37 KB)
