# Supplementary material for: A nationwide cross-sectional analysis of biopsy-proven Fabry nephropathy: the Japan Renal Biopsy Registry
Source: Clin Exp Nephrol. 2022 Nov 3;27(2):141–50. doi: 10.1007/s10157-022-02287-w (PMC9845163; doi:10.1007/s10157-022-02287-w)
Supplement: Supplementary file 1 — Supplementary file1 (DOCX 37 KB) [file 10157_2022_2287_MOESM1_ESM.docx]

**Supplemental appendix**

The following investigators and initial institutions have participated in the development of the J-RBR since 2007: Hirofumi Makino and Hitoshi Sugiyama (Okayama University), late Takashi Taguchi (Nagasaki University), Hitoshi Yokoyama (Kanazawa Medical University), Hiroshi Sato (Tohoku University; present institution: JR Sendai Hospital), Takao Saito (Fukuoka University; present institution: Sanko Clinic), Yoshie Sasatomi (Fukuoka University; present institution: Kanenokuma Hospital), Yukimasa Kohda (Kumamoto University; present institution: Hikarinomori Clinic), Shinichi Nishi (Niigata University; present institution: Kobe University), Kazuhiko Tsuruya (Kyushu University; present institution: Nara Medical University), Yutaka Kiyohara (Kyushu University; present institution: Hisayama Research Institute for Lifestyle Diseases), Hideyasu Kiyomoto (Kagawa University; present institution: Tohoku Medical Megabank Organization, Tohoku University), Hiroyuki Iida (Toyama Prefectural Central Hospital; present institution: Toyama Prefectural Rehabilitation Hospital), Tamaki Sasaki (Kawasaki Medical School),late Makoto Higuchi (Shinshu University), Motoshi Hattori (Tokyo Women’s Medical University), Kazumasa Oka (Osaka Kaisei Hospital; present institution: Hyogo Prefectural Nishinomiya Hospital), Shoji Kagami (The University of Tokushima Graduate School), Michio Nagata (University of Tsukuba), Tetsuya Kawamura (The Jikei University School of Medicine), Masataka Honda (Tokyo Metropolitan Children’s Medical Center), Yuichiro Fukasawa (KKR Sapporo Medical Center; present institution: Sapporo City General Hospital), Atsushi Fukatsu (Kyoto University Graduate School of Medicine; present institution: Fukatsu Medical Clinic), Kunio Morozumi (Japanese Red Cross Nagoya Daini Hospital; present institution: Masuko Memorial Hospital), Norishige Yoshikawa (Wakayama Medical University; present institution: Takatsuki General Hospital), Yukio Yuzawa (Fujita Health University), Seiichi Matsuo (Nagoya University) and Kensuke Joh (Chiba-East National Hospital; present institution: The Jikei University School of Medicine).

# Hokkaido District

・Asahikawa Medical University Hospital (Division of Cardiology, Nephrology, Pulmonology and Neurology, Department of Internal Medicine), Naoki Nakagawa, Motoki Matsuki

・National Hospital Organization Hokkaido Medical Center (Department of Nephrology), Sekiya Shibazaki, Tomotsune Miyamoto, Masanori Ito

・Hokkaido University Graduate School of Medicine (Department of Rheumatology, Endocrinology and Nephrology, Faculty of Medicine and Graduate School of Medicine, Hokkaido University), Saori Nishio, Daigo Nakazawa

・Hokkaido University Graduate School of Medicine (Department of Pediatrics), Takayuki Okamoto, Yasuyuki Sato

・KKR Sapporo Medical Center (Department of Pathology), Akira Suzuki

・Sapporo Medical University (Department of Cardiovascular, Renal and Metabolic Medicine), Tomohisa Yamashita, Marenao Tanaka, Arata Osanami

・Sapporo City General Hospital, Yuichiro Fukasawa

・Teine Keijinkai Hospital (Department of Nephrology), Hideki Takizawa, Norihito Moniwa

# Tohoku District

・Hirosaki University Graduate School of Medicine (Department of Anatomic Pathology), Akira Kurose, (Community Medicine), Michiko Shimada

・Iwate Prefectural Central Hospital (Department of Nephrology and Rheumatology), Jun Soma, Izaya Nakaya

・Fukushima Medical University (Department of Nephrology and Hypertension), Junichiro James Kazama, Kenichi Tanaka, Mizuko Tanaka

・Japan Community Health care Organization Sendai Hospital (Department of Nephrology), Mitsuhiro Sato, Satoru Sanada

・Tohoku University Hospital (Department of Nephrology, Endocrinology, and Hypertension), and affiliated hospitals, Tetsuhiro Tanaka, Mariko Miyazaki, Tasuku Nagasawa, Koji Okamoto

・Yamagata University School of Medicine (Department of Cardiology, Pulmonology, and Nephrology), Tsuneo Konta, Kazunobu Ichikawa

・Yamagata University School of Medicine (Department of Pediatrics), Daisuke Ogino

# Kanto District

・National Hospital Organization Chibahigashi National Hospital (Department of Pathology), Hiroshi Kitamura, (Department of Nephrology), Toshiyuki Imasawa, (Department of Pediatrics), Chieko Matsumura, (Department of Surgery), Naotake Akutsu

・National Hospital Organization Chiba-East Hospital (Department of Urology), Koichi Kamura (*) *present address, Harunclinic Sakura

・Dokkyo Medical University Saitama Medical Center (Department of Nephrology), Tetsuro Takeda

・Dokkyo Medical University (Department of Nephrology and Hypertension ), Toshihiko Ishimitsu

・Gunma University Graduate School of Medicine (Department of Nephrology and Rheumatology), Keiju Hiromura, Yoriaki Kaneko, Hidekazu Ikeuchi, Toru Sakairi

・Jichi Medical University (Division of Nephrology), Daisuke Nagata, Osamu Saito, Tetsu Akimoto

・Jichi Medical University, Saitama Medical Center (Division of Nephrology, Department of Integrated Medicine), Yoshiyuki Morishita

・The Jikei University School of Medicine (Division of Nephrology and Hypertension), Takashi Yokoo, Nobuo Tsuboi, Hiroyuki Ueda, Kentaro Koike, Go Kanzaki

・The Jikei University School of Medicine, Katsushika Medical Center (Division of Nephrology and Hypertension), Yudo Tanno, Shinya Yokote

・The Jikei University School of Medicine, Daisan Hospital (Division of Nephrology and Hypertension), Yoichi Miyazaki, Masahiro Okabe

・The Jikei University Kashiwa Hospital (Division of Nephrology and Hypertension), Masato Ikeda, Akihiro Shimizu

・Juntendo University Faculty of Medicine (Department of Nephrology), Yusuke Suzuki, Tomohito Goda, Masao Kihara, Miyuki Takagi

・Japanese Red Cross Ashikaga Hospital (Department of Nephrology), Keita Hirano

・Kawasaki Municipal Kawasaki Hospital (Division of Nephrology), Takashi Ando, Takahisa Kawaguchi

・Kawaguchi Municipal Medical Center (Division of Nephrology), Masahiro Ishikawa

・Kyorin University School of Medicine (Department of Nephrology and Rheumatology), Shinya Kaname, Kazuhito Fukuoka, Takahisa Kawakami

・Mito Saiseikai General Hospital (Division of Nephrology), Itaru Ebihara, Chihiro Sato

・Nippon Medical School (Division of Nephrology, Department of Internal Medicine), Yukinao Sakai, Akio Hirama, Akiko Mii

・Nihon University School of Medicine (Division of Nephrology, Hypertension and Endocrinology), Seiichiro Hemmi, Masanori Abe

・Saitama Medical University, Faculty of Medicine (Department of Nephrology), Hirokazu Okada, Tsutomu Inoue

・Saitama Medical University, Saitama Medical Center (Department of Nephrology and Hypertension), Takatsugu Iwashita, Akito Maeshima, Hajime Hasegawa

・Saiyu Soka Hospital（Department of Internal Medicine), Masamitsu Ubukata

・Showa University School of Medicine (Division of Nephrology, Department of Medicine), Masayuki Iyoda, Takanori Shibata

・Showa University Fujigaoka Hospital, Internal Medicine Center (Department of Internal Medicine), Yoshihiko Inoue

・St. Marianna University School of Medicine (Division of Nephrology and Hypertension, Department of Internal Medicine), Tomo Suzuki, Daisuke Ichikawa, Sayuri Shirai, Yugo Shibagaki

・Tokai University School of Medicine (Division of Nephrology, Endocrinology and Metabolism), Takehiko Wada, Masafumi Fukagawa

・Teikyo University School of Medicine (Department of Internal Medicine), Yoshihide Fujigaki

・Teikyo University School of Medicine (Department of Urology), Shigeo Horie(*), Satoru Muto(*) *present address, Juntendo University School of Medicine (Department of Urology)

・Tokyo Medical University Ibaraki Medical Center (Department of Nephrology), Masaki Kobayashi, Kouichi Hirayama, Homare Shimohata

・Tokyo Metropolitan Children's Medical Center (Department of Nephrology and Rheumatology), Riku Hamada (Department of General Pediatrics), Hiroshi Hataya

・Tokyo Women's Medical University (Department of Pediatric Nephrology), Motoshi Hattori, Kenichiro Miura, Kiyonobu Ishizuka, Yoko Shirai, Taro Ando

・Tokyo Women's Medical University (Department of Nephrology), Kosaku Nitta, Keiko Uchida, Takahito Moriyama

・Toranomon Hospital, Nephrology Center, Naoki Sawa, Yuki Oba

・The University of Tokyo (Department of Nephrology and Endocrinology), Masaomi Nangaku, Yoshifumi Hamasaki, Yosuke Hirakawa, Ryo Matsuura

・The University of Tokyo(Department of Pediatrics), Yutaka Harita, Shoichiro Kanda, Yuko Kajiho

・University of Tsukuba (Department of Nephrology), Kunihiro Yamagata, Joichi Usui, Toshiaki Usui, Ryoya Tsunoda

・Yokohama City University Graduate School of Medicine (Department of Medical Science and Cardiorenal Medicine), Kouichi Tamura, Hiromichi Wakui, Tomohiko Kanaoka, Kengo Azushima

・Yokohama City University Medical Center, Nobuhito Hirawa, Sanae Saka, Akira Fujiwara

# Koushinetsu District

・Niigata University Graduate School of Medical and Dental Sciences (Kidney Research Center Division of Clinical Nephrology and Rheumatology), Ichiei Narita, Shin Goto, Yumi Itoh, Naofumi Imai

・Shinshu University School of Medicine (Department of Nephrology), Yuji Kamijo, Koji Hashimoto, Akinori Yamaguchi, Harada Makoto

・University of Yamanashi Hospital (Department of Nephrology) Kazuya Takahashi, Kohei Uchimura

# Hokuriku District

・National Hospital Organization Kanazawa Medical Center (Department of Nephrology and Rheumatology), Kiyoki Kitagawa

・Kanazawa Medical University School of Medicine (Department of Nephrology), Hitoshi Yokoyama, Kengo Furuichi, Keiji Fujimoto, Norifumi Hayashi

・Kanazawa Medical University (Department of Diabetology & Endocrinology), Daisuke Koya, Munehiro Kitada, Yuka Kuroshima

・Kanazawa University (Department of Nephrology and Laboratory Medicine), Takashi Wada, Miho Shimizu, Norihiko Sakai, Yasunori Iwata

・Komatsu Sophia Hospital, Yasuhiro Katou, Yuta Yamamura

・Koshino Internal Medicine Clinic, Yoshitaka Koshino

・Public Central Hospital of Matto-Ishikawa, Chikako Takaeda

・Sugita Genpaku Memorial Obama Municipal Hospital, Haruyoshi Yoshida, Takayasu Horiguchi

・Toyama Prefectural Central Hospital (Department of Internal Medicine), Yasuyuki Shinozaki, Masahiko Kawabata

・Toyama City Hospital (Department of Internal Medicine), Satoshi Ota, Yoh-ichi Ishida

・University of Fukui, Faculty of Medical Sciences (Department of Nephrology), Masayuki Iwano, Naoki Takahashi, Kenji Kasuno, Daisuke Mikami

・University of Toyama (Second Department of Internal Medicine), Hidenori Yamazaki

# Tokai District

・Aichi Children's Health and Medical Center (Department of Pediatric Nephrology), Naoya Fujita, Kazuki Tanaka, Reiko Yamaguchi

・Aichi Medical University School of Medicine (Division of Nephrology and Rheumatology), Yasuhiko Ito, Takuhito Nagai, Takayuki Katsuno, Hironobu Nobata

・Chuno Kosei Hospital, Shogo Kimura (*) *present address, Higashiikebukuro Kimura Internal medicine clinic

・Fujinomiya City General Hospital, Masanori Sakakima

・Fujita Health University School of Medicine (Department of Nephrology), Yukio Yuzawa, Naotake Tsuboi, Hiroki Hayashi, Kazuo Takahashi

・Hamamatsu University School of Medicine, University Hospital (Internal Medicine1, Division of Nephrology), Hideo Yasuda, Naro Ohashi, Taichi Sato

・Japanese Red Cross Aichi Medical Center Nagoya Daini Hospital (Nephrology), Aasami Takeda, Hibiki Shinjo

・Nagoya City University East Medical Center, Taisei Suzuki, Minami Mizutani

・Nagoya City University Graduate School of Medical Sciences (Department of Nephrology), Takayuki Hamano, Miho Murashima, Masashi Mizuno

・Nagoya Kyoritsu Hospital (Department of Internal Medicine), Hirotake Kasuga

・Nagoya University Graduate School of Medicine (Department of Nephrology), Shoichi Maruyama, Yoshinari Yasuda, Tomoki Kosugi, Noritoshi Kato

・Shizuoka General Hospital (Department of Nephrology), Noriko Mori, Satoshi Tanaka

・Mie University Graduate School of Medicine (Department of Cardiology and Nephrology), Tomohiro Murata, Yosuke Hirabayashi, Mika Fujimoto, Kan Katayama

・Japan Community Health care Organization Yokkaichi Hazu Medical Center (Division of Nephrology and Blood Purification), Yasuhide Mizutani, Masato Miyake, Shunpei Nawa

# Kinki District

・Hyogo Prefectural Nisihinomiya Hospital (Department of Pathology), Kazumasa Oka

・Hyogo Prefectural Kobe Children's Hospital (Department of Nephrology), Hiroshi Kaito

・Ikeda City Hospital (Department of Nephrology), Nobuyuki Kajiwara

・Kitano Hospital, Tazuke Kofukai Medical Research Institute (Department of Nephrology and Dialysis), Tatsuo Tsukamoto, Tomomi Endo, Keita Mori, Eri Muso

・Kobe University Graduate School of Medicine (Division of Nephrology and Kidney Center), Shinichi Nishi, Shunsuke Goto

・Kobe University Graduate School of Medicine (Department of Pediatrics), Kazumoto Iijima, Kandai Nozu, Tomoko Horinouchi

・Japan Community Health care Organization Kobe Central Hospital, Yoko Adachi, Takaaki Nishihara, Michitsugu Kamezaki

・National Hospital Organization Kyoto Medical Center (Division of Nephrology), Koichi Seta

・Kyoto Prefectural University of Medicine Graduate School of Medical Science (Department of Nephrology), Keiichi Tamagaki, Tetsuro Kusaba, Noriyoshi Ota

・Kyoto University Graduate School of Medicine (Department of Nephrology), Motoko Yanagita, Hideki Yokoi, Kaoru Sakai, Akira Ishii

・Nara Medical University (Department of Nephrology), Kazuhiko Tsuruya, Kenichi Samejima

・National Cerebral and Cardiovascular Center (Division of Hypertension and Nephrology), Fumiki Yoshihara

・National Hospital Organization Osaka National Hospital (Department of Nephrology), Hirotsugu Iwatani

・Osaka City University Graduate School of Medicine (Department of Nephrology), Katsuhito Mori, Akihiro Tsuda, Shinya Nakatani

・Osaka City General Hospital (Division of Nephrology and Hypertension), Yoshio Konishi, Takashi Morikawa, Chizuko Kitabayashi

・Osaka City General Hospital (Division of Pediatrics), Rika Fujimaru

・Osaka General Medical Center (Department of Kidney Disease and Hypertension), Terumasa Hayashi

・Osaka Women's and Children's Hospital (Department of Pediatric Nephrology and Metabolism), Katsusuke Yamamoto

・Osaka Medical and Pharmaceutical University (Department of Pediatrics), Akira Ashida

・Osaka Red Cross Hospital (Department of Nephrology), Yoshihisa Ogawa (*) *present address, Ogawa Clinic

・Osaka Rosai Hospital (Department of Nephrology), Atsushi Yamauchi, Katsuyuki Nagatoya, Daisuke Mori, Ryota Haga

・Osaka University Graduate School of Medicine (Department of Nephrology), Yoshitaka Isaka, Ryohei Yamamoto, Tomoko Namba-Hamano

・Saiseikai Shiga Hospital (Division of Nephrology), Toshiki Nishio

・Shiga University of Medical Science (Department of Medicine), Shinji Kume

・Shirasagi Hospital (Kidney Center), Shigeichi Shoji, Kenjiro Yamakawa, Senji Okuno

・Toyonaka Municipal Hospital (Division of Nephrology), Megumu Fukunaga (*) *present address, Toyonaka Keijinkai Clinic

・Wakayama Medical University (Department of Pediatrics), Yuko Shima,

・Wakayama Medical University (Department of Nephrology), Takashi Shigematsu, Masaki Ohya

# Chugoku District

・Kawasaki Medical School (Department of Nephrology and Hypertension), Naoki Kashihara, Tamaki Sasaki, Hajime Nagasu

・Kurashiki Central Hospital (Division of Nephrology), Kenichiro Asano, Motoko Kanzaki, Kosuke Fukuoka

・Hiroshima University Hospital (Department of Nephrology), Takao Masaki, Ayumu Nakashima, Kensuke Sasaki

・Mizushima Kyodo Hospital (Department of Nephrology), Kan Yamazaki, Nobuyoshi Sugiyama, Yuichiro Inaba, Kouji Ozeki

・Okayama Saiseikai General Hospital (Department of Nephrology), Makoto Hiramatsu, Keisuke Maruyama, Noriya Momoki

・Okayama University Graduate School of Medicine, Dentistry and Pharmaceutical Sciences (Department of Nephrology, Rheumatology, Endocrinology and Metabolism), Jun Wada, Hiroshi Morinaga, Ayu Akiyama, Yasuhiro Onishi

・Okayama University Graduate School of Medicine, Dentistry and Pharmaceutical Sciences (Department of Pediatrics), Hiroyuki Miyahara

・Saiseikai Yamaguchi General Hospital (Department of Internal Medicine), Tsuyoshi Imai

・Shimane University Hospital (Division of Nephrology), Takafumi Ito, Masahiro Egawa, Shohei Fukunaga

・Tottori University, Faculty of Medicine (Division of Pediatrics and Perinatology), Hiroki Yokoyama, Yuko Yamada

・Yonago Medical Center (Department of Pediatrics), Shinichi Okada

# Shikoku District

・Kagawa University, Faculty of Medicine (Department of Cardiorenal and Cerebrovascular Medicine & Department of Clinical Pathology), Tadashi Sofue, Tetsuo Minamino, Emi Ibuki

・Kochi University, Kochi Medical School (Department of Endocrinology, Metabolism and Nephrology), Yoshio Terada, Taro Horino, Satoshi Inotani, Tatsuki Matsumoto

・Kochi University, Kochi Medical School (Department of Pediatrics), Mikiya Fujieda, Masayuki Ishihara, Yoshiki Nagao

・Tokushima University Graduate School (Department of Pediatrics, Institute of Biomedical Sciences), Shoji Kagami, Maki Urushihara, Yukiko Kinoshita

・Tokushima University Graduate School (Department of Nephrology, Institute of Biomedical Sciences), Eriko Shibata, Masanori Tamaki, Kazuhiro Hasegawa, Shu Wakino

# Kyushu District

・Fukuoka University (Division of Nephrology and Rheumatology, Department of Internal Medicine, Faculty of Medicine), Kosuke Masutani, Tetsuhiko Yasuno, Kenji Ito

・Japanese Red Cross Fukuoka Hospital (Department of Pediatrics), Ken Hatae, Mami Washio, Hiroyo Maruyama, Rena Matsuda

・Japanese Red Cross Fukuoka Hospital (Nephrology and Dialysis Center), Koji Mitsuiki (*)*present address, Department of Nephrology, Harasanshin Hospital

・Kumamoto University Graduate School of Medical Sciences (Department of Nephrology), Masashi Mukoyama, Masataka Adachi

・Kurume University School of Medicine (Division of Nephrology, Department of Medicine), Kei Fukami, Junko Yano

・Kyushu University Graduate School of Medical Sciences (Department of Medicine and Clinical Science), Toshiaki Nakano, Akihiro Tsuchimoto, Yuta Matsukuma, Kenji Ueki

・Kyushu University Graduate School of Medical Sciences (Department of Environmental Medicine), Toshiharu Ninomiya, Masaharu Nagata

・Hisayama Research Institute for Lifestyle Diseases, Yutaka Kiyohara

・Miyazaki Prefectural Miyazaki Hospital (Division of Nephrology), Naoko Yokota-Ikeda, Keiko Kodama

・Nagasaki University Hospital (Department of Pathology), late Takashi Taguchi

・Nagasaki University Hospital (Department of Nephrology), Tomoya Nishino, Kumiko Muta, Kenta Torigoe

・National Hospital Organization Fukuokahigashi Medical Center (Division of Nephrology), Yusuke Kuroki

・National Hospital Organization Kyushu Medical Center, Masaru Nakayama

・Oitaken Kouseiren Tsurumi Hospital (Division of Nephrology), Ryokichi Yasumori

・Japanese Red Cross Oita Hospital (Department of Nephrology), Koji Kaneda

・Saga University, Faculty of Medicine (Department of Internal Medicine), Motoaki Miyazono, Makoto Fukuda, Masatora Yamasaki, Yuki Ikeda, Maki Yoshihara

・St. Mary's Hospital, Harumichi Higashi

・University of Miyazaki Hospital (Division of Nephrology), Shouichi Fujimoto, Masao Kikuchi, Shoko Ochiai

・University of Miyazaki (Division of Pediatrics, Department of Developmental and UrologicalReproductive Medicine, Faculty of Medicine), Takao Konomoto, Etsuko Tanaka, Jun Kurogi, Hiromi Sakaguchi

・University of Occupational and Environmental Health (Second Department of Internal Medicine), Masahito Tamura, Tetsu Miyamoto

・University of the Ryukyus Graduate School of Medicine (Department of Cardiology, Nephrology and Neurology), Yusuke Ohya, Kentaro Kohagura

・Okinawa chubu prefectural hospital (Division of Nephrology), Hitoshi Miyasato, Yoshihiko Raita, Kazuki Koga
